# Follicular fluid PlGF and IVF/ICSI outcomes among PCOS and normo-ovulatory women using different controlled hyperstimulation protocols: A prospective case-control study

**DOI:** 10.1016/j.amsu.2022.104096

**Published:** 2022-06-25

**Authors:** Sally Kadoura, Marwan Alhalabi, Abdul Hakim Nattouf

**Affiliations:** a Department of Pharmaceutics and Pharmaceutical Technology, Faculty of Pharmacy, Damascus University, Damascus, Syrian Arab Republic; b Department of Embryology and Reproductive Medicine, Faculty of Medicine, Damascus University, Damascus, Syrian Arab Republic; c Assisted Reproduction Unit, Orient Hospital, Damascus, Syrian Arab Republic

**Keywords:** GnRH antagonist, GnRH agonist, PCOS, IVF, PlGF

## Abstract

**Background:**

Gonadotropin-releasing hormone (GnRH) analogues are used to prevent premature luteinizing hormone (LH) surge during In-Vitro Fertilization. However, the follicular fluid levels of the Placental growth factor (FF PlGF), the novel angiogenic factor, differ significantly between GnRH-agonist and GnRH-antagonist protocols. Thus, we compared the IVF/ICSI outcomes and their correlations with FF PlGF levels in polycystic ovary syndrome (PCOS) and normo-ovulatory women during different hyperstimulation protocols.

**Methods:**

This case-control study is a re-analysis of two prospective trials that were conducted on women who were referred to Orient Hospital, Damascus, Syria, from December 2019 to August 2021. A total of 75 PCOS-women (PCOS-Agonist, n = 53; PCOS-Antagonist, n = 22) and 83 normo-ovulatory women (Control-Agonist, n = 50; Control-Antagonist, n = 33) were included. Follicular fluid samples were collected on retrieval day.

**Results:**

Although PCOS-women were stimulated using lower gonadotropin doses, the Ovarian-sensitivity-indexes were higher in PCOS-groups (PCOS-Agonist vs Control-Agonist; P-value <0.001), (PCOS-Antagonist vs Control-Antagonist; P-value = 0.042). However, FF PlGF levels, maturation rate, fertilization rate, and oocytes morphology were comparable between PCOS and controls independently of the protocol used. Interestingly, FF PlGF levels were positively correlated with Ovarian-sensitivity-indexes in the PCOS-Antagonist, Control-Agonist, and Control-Anta groups, but not in the PCOS-Agonist group. Nevertheless, FF PlGF levels were comparable between pregnant and non-pregnant women in all studied groups.

**Conclusions:**

Although PCOS exaggerates ovarian response to stimulation irrespective of the protocol used, it does not have a detrimental impact on oocytes morphology or competence. Moreover, FF PlGF levels could be a marker of the ovarian response other than a predictor of pregnancy achievement.

## Highlights

• PCOS exaggerates ovarian response to hyperstimulation irrespective of the protocol used.
• The oocytes morphology, quality, and competence are comparable between PCOS and Non-PCOS women.
• FF PlGF levels could be a marker of the ovarian response other than a predictor of pregnancy achievement during IVF/ICSI cycles independent of the PCOS pathology.

## 1 Introduction

Polycystic ovary syndrome (PCOS) is the most common endocrine disorder among females of reproductive age, with a worldwide prevalence of 5–20% [1,2]. In addition, it is considered the main cause of anovulation infertility [3]. In-vitro Fertilization/Intra-Cytoplasmic Sperm Injection (IVF/ICSI) technologies are usually added to the plan-therapy of PCOS women as a third-line treatment choice after the failure of other approaches of ovulation induction [4]. However, since the long Gonadotropin-releasing hormone (GnRH) agonist protocol is still the first-line choice in most of the infertility clinics, only limited and conflicted data are available about the impact of PCOS on the IVF/ICSI outcomes during the GnRH antagonist protocols [[5], [6], [7]]. In addition, no previous study has investigated in detail the impact of PCOS on the oocyte morphology during both the long GnRH agonist protocol and the flexible GnRH antagonist one.

The placental growth factor (PlGF) is an angiogenic growth factor that belongs to the vascular endothelial growth factor (VEGF) family, which contains VEGF-A (also known as VEGF), VEGF-B, VEGF-C, VEGF-D, and VEGF-E, that is known for its role in regulating vasculogenesis and angiogenesis [8]. In addition, recent research has revealed an important role for PlGF in regulating placentation, implantation [[9], [10], [11], [12], [13]], ovarian angiogenesis [14], and ovulation [15]. Besides, imbalance in PlGF levels has been linked to several pregnancy complications like preeclampsia, giving birth of small for gestational age, preterm birth, and stillbirth [[16], [17], [18], [19]]. Although the pathophysiology of PCOS is still not fully understood, growing evidence suggests an important role for angiogenic dysregulation [20]. PCOS ovaries exhibit higher vascularization and lower impedance to flow in ovarian stromal vessels compared to control [[21], [22], [23]]. Although data are inconsistence, some reports showed that PCOS women have higher levels of the pro-angiogenic factor, VEGF, and lower levels of the anti-angiogenic factor, soluble form of VEGF receptor-1 (sVEGFR-1 or also known as soluble Fms-like tyrosine kinase-1, sFlt-1), compared to controls both in serum and follicular fluid samples [24,25]. Nevertheless, since VEGF is considered the main member of the VEGF family, most of the available research on angiogenesis were interested in detecting its role in PCOS pathology more than the roles of the other VEGF family members. To our best knowledge, only one study [26] compared the follicular fluid levels of placental growth factor (FF PlGF) between 14 PCOS and 14 control women, and it declared higher FF PlGF levels in PCOS subjects. However, that study used a combination of GnRH agonist and GnRH antagonist protocols. Based on our recent work, FF PlGF levels differ significantly between the long GnRH agonist and the flexible GnRH antagonist protocols both; in PCOS and normo-ovulatory women [27,28]. Thus, it is unclear whether these differences in FF PlGF levels between PCOS and controls would still be important after adjusting to the type of protocol used. In addition, no previous study has investigated the correlations between FF PlGF levels and IVF/ICSI outcomes in PCOS women, and no similar study has been conducted on non-PCOS women during the GnRH antagonist protocol. Moreover, the only study [29] that was done on the non-PCOS population during the long agonist protocol included various types of ovarian response; poor responders, normo-responders, and high responders. Thus, we conducted this study, which aimed to compare the IVF/ICSI outcomes and their correlations with FF PlGF levels in polycystic ovary syndrome women and normo-ovulatory women during the long GnRH agonist protocol and the flexible GnRH antagonist protocol.

## 2 Material and methods

### 2.1 Study design

The case-control study is a re-analysis of our previous work. The data were adopted from two prospective clinical trials [27,28] that were registered on the clinicaltrials.gov site on registration numbers NCT04727671 (https://clinicaltrials.gov/ct2/show/NCT04727671) and NCT04724343 (https://clinicaltrials.gov/ct2/show/NCT04724343). The trials were conducted on women who were referred to the Assisted Reproductive Unit of Orient Hospital, Damascus, Syrian Arab Republic, from December 2019 to August 2021. Orient Hospital is a professional unit affiliated to the Faculty of Medicine, Damascus University, and an approved training facility for Assisted Reproduction Technology (ART) by the Ministry of Health in Syria. The studies were performed in line with the principles of the Declaration of Helsinki. The Ethical Committee of Damascus University approved the studies’ protocols, and a written informed consent was obtained from all participants. The study was reported according to STROCSS guidelines (2021) [30].

### 2.2 Participants

A total of 75 PCOS women (GnRH agonist group, PCOS-A, n = 53; GnRH antagonist group, PCOS-Anta, n = 22) and 83 normo-ovulatory women (GnRH agonist group, Control-A, n = 50; GnRH antagonist group, Control-Anta, n = 33) were included. Both the patients and the doctors were aware of the allocated arm. PCOS diagnosed was according to the Rotterdam criteria [31]; the presence of at least two of the following three criteria: (1) oligo or anovulation, (2) clinical and/or biochemical signs of hyperandrogenism, (3) polycystic ovarian morphology on ultrasound examination (defined as the presence of 12 or more follicles in each ovary measuring 2–9 mm in diameter and/or an ovarian volume >10 ml) with the exclusion of other possible etiologies. The control groups included women that undergone IVF/ICSI cycles due to male or tubal factors. The exclusion criteria for all participants were patients who aged ≥40 years; or those diagnosed with androgen-secreting tumors, Cushing's syndrome, congenital adrenal hyperplasia, hyperprolactinemia, thyroid disorders, epilepsy, diabetes mellitus, cardiovascular diseases, liver diseases, kidney diseases, cancer; or had any conditions that might affect IVF outcomes like endometriosis, uterine fibroids, hydrosalpinx, adenomyosis, or autoimmune diseases. Women with three or more previous IVF failures, poor responders (Bologna criteria [32]), and those who were previously undergone unilateral oophorectomy were also excluded.

### 2.3 Controlled ovarian stimulation protocols

#### 2.3.1 Agonist groups (long protocol)

The pituitary down-regulation in this group was carried out using 0.05–0.1 mg of Triptorelin acetate subcutaneously (SC) once daily from the mid-luteal phase (day 21) of the menstrual cycle until the ovulation triggering day. When the suppressive effect was obtained (Estradiol <50 pg/ml, no cysts or follicles >1 cm maximum diameter detected by ultrasound, endometrial thickness <5 mm), ovarian stimulation was commenced with recombinant Follicle-Stimulating Hormone (r-FSH) and/or human Menopausal Gonadotropin (hMG), and the dose was adjusted according to the ovarian response, which was monitored by transvaginal ultrasound (Voluson TM E10, GE Healthcare Ultrasound, USA).

#### 2.3.2 Antagonist groups (conventional flexible protocol)

The ovarian stimulation in this group was started with recombinant Follicle-Stimulating Hormone (r-FSH) and/or human Menopausal Gonadotropin (hMG) on the third day of the menstrual cycle, and the dose was adjusted according to the ovarian response, which was monitored by transvaginal ultrasound (Voluson TM E10, GE Healthcare Ultrasound, USA). The initiation of 0.25 mg of GnRH antagonist, Cetrorelix, took place after detecting a leading follicle diameter ≥14 mm and continued till the day of ovulation triggering.

### 2.4 Ovulation triggering and oocytes retrieval

Ovulation was triggered by the administration of 10,000 IU of human Chorionic Gonadotropin (hCG) when at least three follicles become more than 16–17 mm. After 35 ± 2 h of ovulation triggering, the oocytes were retrieved by transvaginal ultrasound-guided follicle aspiration.

### 2.5 IVF procedure and embryological outcomes assessment

An Intra-Cytoplasmic Sperm Injection (ICSI) technique was used for insemination. The embryological outcomes were assessed by independent highly-trained embryologists. Each studied outcome was assessed by a single assessor for all groups to limit inter-assessor variations. The same media and culturing methodology were used for all groups. The Thermo Scientific HERACELL 150i incubator (Thermo Fisher Scientific, USA) was used for COCs (Cumulus oocyte complex) and oocytes cultures (humidified atmosphere at 37 °C, CO2 level at approximately 6%, and culture medium pH between 7.28 and 7.35), and the K-Systems G210 InviCell (K-Systems Kivex Biotec Ltd. Denmark) was used for Embryos cultures.

#### 2.5.1 Oocyte's denudation and maturation assessment

Retrieved oocytes were first rinsed in G-MOPS ™ Plus media (G-MOPS ™ Plus, Vitrolife, Sweden) then maintained in G-IVF ™ Plus culture (G-IVF ™ Plus, VitroLife, Sweden) covered with paraffin oil (OVOIL, VitroLife, Sweden) before cumulus cell removal. The surrounding cumulus cells were removed within 2 h after retrieval by the exposure to hyaluronidase (HYASE-10 × in G-Mops ™ Plus media, Vitrolife, Sweden) for several seconds before being transferred to G-MOPS ™ Plus media where they were mechanically dissociated from the oocyte.

The denuded oocytes were classified according to their level of maturation using a Nikon SMZ1500 stereoscope. The number of Metaphase II Oocytes (MII; identified as oocytes with the extrusion of the first polar body), Metaphase I Oocytes (MI; identified as oocytes lack the presence of both the germinal vesicle and the polar body), Germinal Vesicle Oocytes (GV; identified as oocytes with Germinal Vesicle), and Atretic Oocytes (oocytes with signs of degeneration) were documented. The Maturation Rate was calculated by dividing the number of mature (MII) oocytes by the number of retrieved oocytes. In addition, the ovarian sensitivity index (OSI) was calculated by dividing the number of retrieved oocytes by the total dose of FSH used and multiplying the results by 1000 [33].

#### 2.5.2 Oocytes morphological assessment

Before being subjected to ICSI, MII oocytes from both groups were morphologically assessed using an inverted microscope Nikon Eclipse Ti2 (Nikon, Tokyo, Japan) under 400 × magnification. The following dysmorphisms were studied:

• Cytoplasmic dysmorphisms: the presence of granulation, refractile bodies, smooth endoplasmic reticulum (SER) aggregations or vacuoles in the cytoplasm; or detecting dark cytoplasm.
• Extracytoplasmic dysmorphisms:
  • Alterations in oocyte shape or size.
  • Zona pellucida dysmorphisms: alterations in zona pellucida color, size, or thickness; the presence of a zona pellucida with a septum.
  • Perivitelline space dysmorphisms: alterations in perivitelline space size or presence of perivitelline space fragments.
  • Polar body dysmorphisms: alterations in polar body size, presence of polar body fragments, or presence of duplicated/triplicated polar body.

The oocytes were classified as normal oocytes, oocytes with cytoplasmic dysmorphisms, oocytes with extracytoplasmic dysmorphisms, and oocytes with both cytoplasmic and extracytoplasmic dysmorphisms. In addition, the oocytes were classified based on the quantity of the dysmorphisms observed.

#### 2.5.3 Insemination and fertilization assessment

Microinjections were performed at ×400 magnification on a 37 °C heated stage inverted Nikon Eclipse Ti2 (Nikon, Tokyo, Japan). A Petri dish containing a microdroplet of ICSI ™ media in the center (ICSI ™, VitroLife, Sweden) under paraffin oil (OVOIL, VitroLife, Sweden) was used for sperms selection and immobilization. On the same dish, a microdroplet of G-Gamete ™ culture medium (G-Gamete ™, Vitrolife, Sweden) was used for placing the oocytes for microinjection. A single sperm was mechanically immobilized using the tip of the microinjection needle (Origio, USA) and then was aspirated inside the needle. The oocyte was held in place using a 35-degree angle holding micropipette (Origio, USA) with the polar body in the 6 or 12 o'clock position. Injection of a single spermatozoon within the oocyte cytoplasm was performed by using a micromanipulator (TransferMan® 4r, eppendorf, Germany). After ICSI, injected oocytes were cultured in G1-Plus ™ medium (G1-Plus ™, VitroLife, Sweden). Fertilization was confirmed by the presence of two pronuclei and the extrusion of the second polar body approximately 16–18 h after ICSI. The Fertilization Rate was calculated by dividing the number of obtained zygotes (2 PN) by the number of injected oocytes.

#### 2.5.4 Embryos grading, cleavage rate, and high-quality embryos rate

Embryos were morphologically evaluated using Nikon SMZ1500 stereoscope microscope (Nikon, Tokyo, Japan) and were graded based on ESHRE criteria (2011) [34]. According to these criteria, high-quality cleavage-stage embryos are defined as those with all of the following characteristics: 2–4 cells on day 2 or 6–8 cells on day 3, <10% fragmentation, symmetric blastomeres, and absence of multinucleation. Cleavage rate was calculated by dividing the number of cleavaged embryos by the number of obtained zygotes (2 PN), while High-Quality Embryos Rate was calculated by dividing the number of high-quality embryos (Grade I) obtained by the total number of cleavaged embryos obtained.

#### 2.5.5 Embryos transfer and luteal phase support

The Selected embryos were treated with EmbryoGlue® media (EmbryoGlue®, VitroLife, Sweden) before being transferred using a Sure-Pro Ultra catheter (Wallace, USA) under transvaginal ultrasound guidance on day 2–3 after insemination (cleavage stage embryos). Luteal phase support was achieved using vaginal micronized progesterone gel (Crinone ® 8%, 10.13039/100004334Merck Serono). It was started from the day of oocyte retrieval and continued for 14 days when a pregnancy was carried out. If pregnancy was confirmed, progesterone administration was continued until the 12th week of pregnancy.

Embryo transfer was cancelled, and elective embryo cryopreservation was performed in cases that were highly suspected of developing life-threatening (critical) OHSS [35,36] or fulfill the criteria for OHSS hospitalization [37]. Cycle Cancellation Rate (CCR) was calculated by dividing the number of cycle cancellation cases by the total number of participants.

### 2.6 Follicular fluid collection and analysis

Follicular fluid was aspirated from all follicles (>15) mm, and then it was centrifuged at 3000 g for 10 min at room temperature, and the supernatant was stored at −80 °C until assayed. Follicular fluid concentrations of Anti-Müllerian hormone (AMH) were assayed using an ELISA kit from Biorex diagnostics (United Kingdom). Follicular fluid concentrations of PlGF were assayed using an ELISA kit from DRG Instruments (Germany). The intra-assay and inter-assay coefficients of variation for all assays were less than 5% and less than 10%, respectively.

### 2.7 Pregnancy assessment and follow up

A serum pregnancy test (serum hCG) was performed 14 days after embryo transfer. All women with a positive test received a transvaginal ultrasound scan after one-two weeks (i.e., 3–4 weeks after embryo transfer) then followed up until week 12 of gestation. The following rates were calculated:

• Biochemical Pregnancy Rate (BPR): Biochemical pregnancy was defined as a positive serum beta-hCG pregnancy test after two weeks of embryo transfer [38]. BPR was calculated by dividing the number of women who were biochemically pregnant by the total number of participants (Per Woman) or the total number of women who had at least one embryo transferred (Per Embryo Transfer).
• Clinical Pregnancy Rate (CPR): Clinical pregnancy was defined as the presence of at least one gestational sac on ultrasound after 3–4 weeks of embryo transfer. In addition to intra-uterine pregnancy, it included a clinically documented ectopic pregnancy [38]. CPR was calculated by dividing the number of women who were clinically pregnant by the total number of participants (Per Woman) or the total number of women who had at least one embryo transferred (Per Embryo Transfer).
• Multiple Pregnancy Rate (MPR): MPR was calculated by dividing the number of pregnancies with two or more gestational sacs on ultrasound by the total number of participants (Per Woman) or the total number of women who had at least one embryo transferred (Per Embryo Transfer).
• Implantation Rate (IR): IR was calculated by dividing the number of gestational sacs observed by the number of embryos transferred.
• Ongoing Pregnancy Rate (OPR): Ongoing pregnancy was defined as a pregnancy that continued ≥12 weeks of gestation. OPR was calculated by dividing the number of ongoing pregnancies by the total number of participants (Per Woman) or the total number of women who had at least one embryo transferred (Per Embryo Transfer).
• Resolved Pregnancy of unknown location (RPUL) Rate: RPUL was defined as a pregnancy demise not visualized on transvaginal ultrasound with a resolution of serum β-hCG after expectant management or after uterine evacuation without chorionic villi on histology [39]. RPUL Rate was calculated by dividing the number of RPUL cases by the total number of participants (Per Woman) or the total number of women who had at least one embryo transferred (Per Embryo Transfer).

### 2.8 Statistical analysis

All statistical analyses were performed using a Statistical Package for the Social Sciences (SPSS) software version 24.0 (IBM Corp., Armonk, NY, USA). Continuous variables were expressed as mean ± standard deviation and categorical variables as counts with percentages. Between-group comparisons were performed using the independent *t*-test for normally distributed variables, the Mann–Whitney *U* test for non-normally distributed variables, and chi-square or Fisher's exact test as appropriate for categorical variables. Spearman rank correlation coefficients were computed to assess the correlation among the studied parameters. The area under the receiver operating characteristic (ROC) curve (AUC) was used to evaluate the accuracy of follicular fluid PlGF levels in predicting pregnancy rates. For testing all hypotheses, tests were two-tailed, and values less than 0.05 were considered statistically significant.

## 3 Results

There were not any significant differences between PCOS-A and Control-A groups or PCOS-Anta and Control-Anta groups in Female age, male age, infertility history, or other baseline characteristics, as shown in Table 1. Although similar stimulation durations were noted in PCOS groups and control ones, PCOS women were stimulated using lower starting and total doses of gonadotropins (starting dose; PCOS-A = 227.83 ± 72.00 IUs vs Control-A = 294.00 ± 106.97 IUs; P-value = 0.001; Table 2), (starting dose; PCOS-Anta = 225.00 ± 83.45 IUs vs Control-Anta = 331.82 ± 107.76 IUs; P-value <0.001; Table 2), (Total gonadotropins dose; PCOS-A = 1868.40 ± 668.29 IUs vs Control-A = 2523.00 ± 1034.11 IUs; P-value <0.001; Table 2), (Total gonadotropins dose; PCOS-Anta = 1779.55 ± 702.87 IUs vs Control-Anta = 2468.18 ± 879.53 IUs; P-value = 0.003; Table 2). In addition, the number of retrieved, MII, MI, and immature oocytes were significantly higher in the PCOS-A group compared to the Control-A group (retrieved oocytes; PCOS-A = 20.04 ± 9.42 oocytes vs Control-A = 15.46 ± 6.05 oocytes; P-value = 0.014; Table 3), (MII oocytes; PCOS-A = 11.89 ± 5.65 oocytes vs Control-A = 9.08 ± 4.50 oocytes; P-value = 0.006; Table 3), (MI oocytes; PCOS-A = 4.19 ± 3.23 oocytes vs Control-A = 2.76 ± 1.57 oocytes; P-value = 0.005; Table 3), (immature oocytes; PCOS-A = 7.28 ± 4.55 oocytes vs Control-A = 5.50 ± 3.09 oocytes; P-value = 0.023; Table 3). The number of fertilized oocytes and obtained embryos were also higher in the PCOS-A group compared to the Control-A group, but the results did not reach the significance level. On the other hand, similar effects were not noted between PCOS and controls during the GnRH antagonist protocol. Nevertheless, OSI values were significantly higher in PCOS groups independently of the protocol used (OSI; PCOS-A = 11.83 ± 6.98 Oocyte/IU vs Control-A = 7.48 ± 4.75 Oocyte/IU; P-value <0.001; Table 3), (OSI; PCOS-Anta = 11.46 ± 8.99 Oocyte/IU vs Control-Anta = 7.37 ± 4.87 Oocyte/IU; P-value = 0.042; Table 3). On the other hand, there were no significant differences between PCOS and controls in maturation rate, fertilization rate, high-quality embryos rate, cleavage rate, implantation rate or oocytes morphology during both protocols as shown in Table 3, Table 4. Follicular fluid levels of AMH were significantly higher in the PCOS-A group compared to the Control-A one (FF AMH; PCOS-A = 13.62 ± 15.25 ng/ml vs Control-A = 7.40 ± 5.69; P-value = 0.006; Table 3), while FF PlGF levels did not differ significantly between the two groups. Similar effects were also noted during the GnRH antagonist protocol (FF AMH; PCOS-Anta = 16.93 ± 18.08 ng/ml vs Control-Anta = 8.51 ± 7.93; P-value = 0.036; Table 3). On the other hand, there were not any significant differences between the PCOS groups and the control groups in clinical IVF/ICSI outcomes independently of the protocol used as shown in Table 4. Regarding correlations between FF PlGF and IVF/ICSI outcomes, FF PlGF levels were negatively correlated with age and total gonadotropins dose and positively correlated with OSI in the PCOS-Anta (age, r = −0.435, P = 0.043; total gonadotropins dose, r = −0.467, P = 0.029; OSI, r = 0.428, P = 0.047; Table 5), Control-A (age, r = −0.328, P = 0.020; total gonadotropins dose, r = −0.431, P = 0.002; OSI, r = 0.447, P = 0.001; Table 5), and Control-Anta groups (age, r = −0.361, P = 0.039; total gonadotropins dose, r = −0.478, P = 0.005; OSI, r = 0.359, P = 0.040; Table 5), but not in the PCOS-A group. Moreover, FF PlGF levels positively correlated with the number of MII oocytes in the PCOS-Anta group (r = 0.500, P = 0.018; Table 5) and the number of retrieved oocytes in the Control-A group (r = 0.316, P = 0.026; Table 5), while a positive correlation between FF PlGF and gonadotropins starting dose was noted in Control-A (r = −0.446, P = 0.001; Table 5) and Control-Anta (r = −0.464, P = 0.007; Table 5) groups. Nevertheless, no significant differences were noted in FF PlGF levels between pregnant and non-pregnant women in any of the studied groups, as shown in Table 6, which also was confirmed by the Receiver Operating Characteristic (ROC) Curve analysis (Table 7).

**Table 1:** Patients baseline characteristics.

	PCOS		Controls		P-Value§	P-Value‡
	GnRH Agonist N = 53	GnRH Antagonist N = 22	GnRH Agonist N = 50	GnRH Antagonist N = 33		
Female age (years)	27.87 ± 4.57	27.09 ± 5.15	28.12 ± 5.30	28.88 ± 6.29	0.796	0.274
Male age (years)	35.51 ± 6.41	34.64 ± 6.96	36.88 ± 7.13	37.58 ± 8.84	0.394	0.196
Infertility % (n)						
Primary	67.9% (36/53)	54.5% (12/22)	74.0% (37/50)	78.8% (26/33)	0.498	0.057
Secondary	32.1% (17/53)	45.5% (10/22)	26.0% (13/50)	21.2% (7/33)		
Infertility duration (years)	5.75 ± 3.35	6.25 ± 4.62	6.93 ± 4.06	5.54 ± 3.80	0.110	0.783
Smoker Female % (n)	18.9% (10/53)	36.4% (8/22)	16.0% (8/50)	24.2% (8/33)	0.702	0.332
Smoker Male % (n)	50.9% (27/53)	63.6% (14/22)	44.0% (22/50)	63.6% (21/33)	0.481	1.000
Female alcohol-consuming % (n)	0.0% (0/53)	0.0% (0/22)	0.0% (0/50)	0.0% (0/33)	–	–
Male alcohol-consuming % (n)	1.9% (1/53)	0.0% (0/22)	0% (0/50)	6.1% (2/33)	1.000	0.511
Male classification % (n)						
Normozoospermia	13.2% (7/53)	36.4% (8/22)	10.0% (5/50)	27.3% (9/33)	0.562	0.831
Mild-Moderate Male factor	32.1% (17/53)	31.8% (7/22)	24.0% (12/50)	21.2% (7/33)		
Oligoasthenoteratozoospermia	32.1% (17/53)	18.2% (4/22)	46.0% (23/50)	24.2% (8/33)		
Azoospermia	15% (8/53)	9.1% (2/22)	8.0% (4/50)	12.1% (4/33)		
Necrozoospermia	3.8% (2/53)	0.0% (0/22)	4.0% (2/50)	3.0% (1/33)		
Cryptozoospermia	3.8% (2/53)	4.5% (1/22)	8.0% (4/50)	12.1% (4/33)		

PCOS: Polycystic Ovary Syndrome, P Value§: PCOS-A VS Control-A, P Value‡: PCOS-Anta VS Control-Anta.

**Table 2:** Cycle characteristics.

	PCOS		Controls		P-Value§	P-Value‡
	GnRH Agonist N = 53	GnRH AntagonistN = 22	GnRH AgonistN = 50	GnRH AntagonistN = 33		
FSH starting dose (units)	227.83 ± 72.00	225.00 ± 83.45	294.00 ± 106.97	331.82 ± 107.76	**0.001**	**<0.001**
Total FSH dose (units)	1868.40 ± 668.29	1779.55 ± 702.87	2523.00 ± 1034.11	2468.18 ± 879.53	**<0.001**	**0.003**
Stimulation duration (days)	8.04 ± 0.81	7.64 ± 1.22	8.28 ± 1.09	7.26 ± 0.89	0.264	0.364
Sperms Source % (n)						
Ejection	75.5% (40/53)	77.3% (17/22)	70.0% (35/50)	81.8% (27/33)	0.675	0.737
Tesa	22.6% (12/53)	9.1% (2/22)	22.0% (11/50)	12.1% (4/33)		
Pesa	0.0% (0/53)	0.0% (0/22)	4.0% (2/50)	0.0% (0/33)		
Frozen	0.0% (0/53)	4.5% (1/22)	2.0% (1/50)	0.0% (0/33)		
Ejection + Tesa	1.9% (1/53)	9.1% (2/22)	2.0% (1/50)	6.1% (233/)		
Day of transfer						
Day 2	63.3% (31/49)	57.9% (11/19)	76.6% (36/47)	77.4% (24/31)	0.144	0.155
Day 3	36.7% (18/49)	42.1% (8/19)	23.4% (11/47)	22.6% (7/31)		
Cycle cancellation Rate % (n)	7.5% (4/53)	13.6% (3/22)	6.0% (3/50)	6.1% (2/33)	1.000	0.379
Cycle cancellation Rate due to risk of OHSS % (n)	5.7% (3/53)	4.5% (1/22)	0.0% (0/50)	0.0% (0/33)	0.243	0.400

FSH: Follicle-Stimulating Hormone, OHSS: Ovarian Hyperstimulation Syndrome, PCOS: Polycystic Ovary Syndrome, Pesa: Percutaneous Epididymal Sperm Aspiration, Tesa: Testicular Sperm Aspiration, P Value§: PCOS-A VS Control-A, P Value‡: PCOS-Anta VS Control-Anta.

**Table 3:** Embryological IVF/ICSI Outcomes and oocyte morphology assessment.

	PCOS		Controls		P-Value§	P-Value‡
	GnRH Agonist N = 53	GnRH Antagonist N = 22	GnRH Agonist N = 50	GnRH Antagonist N = 33		
Number of Retrieved Oocytes	20.04 ± 9.42	17.73 ± 9.76	15.46 ± 6.05	16.24 ± 8.99	**0.014**	0.547
Ovarian Sensitivity Index	11.83 ± 6.98	11.46 ± 8.99	7.48 ± 4.75	7.37 ± 4.87	**<0.001**	**0.042**
Number of Metaphase II Oocytes	11.89 ± 5.65	10.18 ± 5.55	9.08 ± 4.50	9.03 ± 5.34	**0.006**	0.317
Number of Metaphase I Oocytes	4.19 ± 3.23	3.55 ± 2.74	2.76 ± 1.57	3.18 ± 2.02	**0.005**	0.726
Number of GV Stage Oocytes	3.09 ± 2.31	2.5 ± 3.52	2.74 ± 2.31	3.33 ± 4.09	0.421	0.269
Number of Immature Oocytes (GV + MI)	7.28 ± 4.55	6.05 ± 5.63	5.50 ± 3.09	6.52 ± 4.93	**0.023**	0.545
Number of Atretic Oocytes	0.87 ± 1.84	1.5 ± 2.76	0.88 ± 1.84	0.70 ± 1.67	0.984	0.563
Number of Fertilized Oocytes	7.42 ± 4.17	6.73 ± 4.78	5.88 ± 3.87	5.30 ± 3.50	0.068	0.330
Maturation Rate (%)	61.39 ± 14.30	60.13 ± 23.79	58.59 ± 21.12	56.81 ± 18.09	0.552	0.559
Fertilization Rate (%)	63.55 ± 23.55	69.18 ± 31.72	65.63 ± 28.41	59.75 ± 25.41	0.479	0.123
Number of Embryos Obtained	7.32 ± 4.07	6.68 ± 4.82	5.88 ± 3.87	5.30 ± 3.50	0.074	0.225
High-quality Embryos Rate (%)	56.83 ± 23.85	60.41 ± 30.62	60.26 ± 26.25	59.76 ± 26.23	0.395	0.691
Cleavage Rate (%)	97.61 ± 13.92	89.39 ± 29.79	94.00 ± 23.99	96.97 ± 17.41	0.903	0.147
Number of Embryos Transferred	4.47 ± 1.93	3.45 ± 2.41	4.32 ± 1.85	3.76 ± 1.90	0.615	0.708
FF AMH ng/ml	13.62 ± 15.25	16.93 ± 18.08	7.40 ± 5.69	8.51 ± 7.93	**0.006**	**0.036**
FF PlGF pg/ml	142.75 ± 51.48	117.70 ± 35.86	140.46 ± 42.44	120.49 ± 35.07	0.751	0.559
Oocytes Morphology % (n)						
Normal	77.3% (41/53)	77.3% (17/22)	76.0% (38/50)	78.8% (26/33)	0.383	1.000
Cytoplasmic Dysmorphisms	18.9% (10/53)	18.2% (4/22)	20.0% (10/50)	18.2% (6/33)		
Extra-Cytoplasmic Dysmorphisms	0.0% (0/53)	4.5% (1/22)	4.0% (2/50)	3.0% (1/33)		
Both	3.8% (2/53)	0.0% (0/22)	0.0% (0/50)	0.0% (0/33)		
Quantity of oocytes dysmorphisms % (n)						
Normal	77.3% (41/53)	77.3% (17/22)	76.0% (38/5)	78.8% (26/33)	0.935	0.846
One	17.0% (9/53)	22.7% (5/22)	20.0% (10/50)	18.2% (6/33)		
Multi	5.7% (3/53)	0.0% (0/22)	4.0% (2/50)	3.0% (1/33)		
Granulation % (n)	17% (9/53)	9.1% (2/22)	16.0% (8/50)	15.2% (5/33)	0.893	0.689
Refractile Bodies % (n)	0.0% (0/53)	0.0% (0/22)	0.0% (0/50)	0.0% (0/33)	–	–
SER % (n)	0.0% (0/53)	0.0% (0/22)	0.0% (0/50)	0.0% (0/33)	–	–
Vacuoles % (n)	5.7% (3/53)	9.1% (2/22)	6.0% (3/50)	3.0% (1/33)	1.000	0.557
Dark Cytoplasm % (n)	1.9% (1/53)	0.0% (0/22)	2.0% (1/50)	3.0% (1/33)	1.000	1.000
Oocytes Shape % (n)	0.0% (0/53)	4.5% (1/22)	0.0% (0/50)	0.0% (0/33)	–	0.400
Oocytes Size % (n)	0.0% (0/53)	0.0% (0/22)	0.0% (0/50)	0.0% (0/33)	–	–
ZP Dysmorphisms % (n)	0.0% (0/53)	0.0% (0/22)	0.0% (0/50)	0.0% (0/33)	–	–
PVS Dysmorphisms % (n)	0.0% (0/53)	0.0% (0/22)	0.0% (0/50)	0.0% (0/33)	–	–
PB Dysmorphisms % (n)	3.8% (2/53)	0.0% (0/22)	4.0% (2/50)	3.0% (1/33)	1.000	1.000
(Duplicated/Triplicated PB)						

AMH: Anti-Müllerian Hormone, GV: Germinal Vesicle, PB: Polar Body, PCOS: Polycystic Ovary Syndrome, PlGF: Placental Growth Factor, PVS: Perivitelline Space, SER: Smooth Endoplasmic Reticulum Aggregations, ZP: Zona Pellucida, P Value§: PCOS-A VS Control-A, P Value‡: PCOS-Anta VS Control-Anta.

**Table 4:** Clinical IVF/ICSI outcomes.

	PCOS		Controls		P-Value§	P-Value‡
	GnRH Agonist N = 53	GnRH Antagonist N = 22	GnRH Agonist N = 50	GnRH Antagonist N = 33		
Endometrial thickness on hCG day (mm)	9.63 ± 1.19	9.71 ± 1.37	9.66 ± 1.39	9.03 ± 1.51	0.891	0.101
Biochemical Pregnancy Rate % (n)						
Per Woman:	43.4% (23/53)	36.4% (8/22)	36.0% (18/50)	30.3% (10/33)	0.443	0.639
Per Embryo Transfer:	46.9% (23/49)	42.1% (8/19)	38.3% (18/47)	32.3% (10/31)	0.392	0.481
Clinical Pregnancy Rate % (n)						
Per Woman:	39.6% (21/53)	36.4% (8/22)	30.0% (15/50)	27.3% (9/33)	0.306	0.475
Per Embryo Transfer:	42.9% (21/49)	42.1% (8/19)	31.9% (15/47)	29.0% (9/31)	0.268	0.344
Ongoing Pregnancy Rate % (n)						
Per Woman:	32.1% (17/53)	36.4% (8/22)	24.0% (12/50)	24.2% (8/33)	0.362	0.332
Per Embryo Transfer:	34.7% (17/49)	42.1% (8/19)	25.5% (12/47)	25.8% (8/31)	0.328	0.230
Multiple Pregnancy Rate % (n)						
Per Woman:	17% (9/53)	22.7% (5/22)	14.0% (7/50)	12.1% (4/33)	0.676	0.459
Per Embryo Transfer:	18.4% (9/49)	26.3% (5/19)	14.9% (7/47)	12.9% (4/31)	0.648	0.273
Implantation Rate %	15.06 ± 22.86	18.05 ± 31.14	9.43 ± 16.02	10.81 ± 20.59	0.251	0.456
Resolved PUL% (n)						
Per Woman:	3.8% (2/53)	0.0% (0/22)	4.0% (2/50)	3.0% (1/33)	1.000	1.000
Per Embryo Transfer:	4.1% (2/49)	0.0% (0/19)	4.3% (2/47)	3.2% (1/31)	1.000	1.000
Ectopic Pregnancy Rate % (n)						
Per Woman:	3.8% (2/53)	0.0% (0/22)	0.0% (0/50)	0.0% (0/33)	0.496	–
Per Embryo Transfer:	4.1% (2/49)	0.0% (0/19)	0.0% (0/47)	0.0% (0/31)	0.495	–
Hospitalized OHSS Rate % (n)	3.8% (2/53)	4.5% (1/22)	0.0% (0/50)	0.0% (0/33)	0.496	0.400

hCG: human Chorionic Gonadotropin, OHSS: Ovarian Hyperstimulation Syndrome, PCOS: Polycystic Ovary Syndrome, PUL: Pregnancy of Unknown Location, P Value§: PCOS-A VS Control-A, P Value‡: PCOS-Anta VS Control-Anta.

**Table 5:** Correlations between FF PlGF and IVF/ICSI outcomes.

	PCOS				Controls			
	GnRH Agonist N = 53		GnRH Antagonist N = 22		GnRH Agonist N = 50		GnRH Antagonist N = 33	
	Correlation Coefficient	P-value	Correlation Coefficient	P-value	Correlation Coefficient	P-value	Correlation Coefficient	P-value
FF AMH ng/ml	0.165	0.238	0.252	0.258	0.206	0.151	0.193	0.283
Female age (years)	−0.105	0.453	**−0.435**	**0.043**	**−0.328**	**0.020**	**−0.361**	**0.039**
Infertility duration (years)	−0.008	0.952	−0.235	0.293	−0.144	0.319	−0.122	0.499
FSH starting dose (units)	−0.009	0.951	−0.344	0.117	**−0.446**	**0.001**	**−0.464**	**0.007**
Total FSH dose (units)	−0.037	0.793	**−0.467**	**0.029**	**−0.431**	**0.002**	**−0.478**	**0.005**
Stimulation duration (days)	0.025	0.857	−0.236	0.290	−0.240	0.093	−0.268	0.131
Endometrial thickness on hCG day (mm)	−0.046	0.746	0.239	0.284	−0.176	0.221	0.260	0.144
Number of Retrieved Oocytes	−0.001	0.997	0.302	0.172	**0.316**	**0.026**	0.171	0.341
Ovarian Sensitivity Index	−0.012	0.929	**0.428**	**0.047**	**0.447**	**0.001**	**0.359**	**0.040**
Number of Metaphase II Oocytes	0.006	0.965	**0.500**	**0.018**	0.178	0.215	0.110	0.541
Number of Metaphase I Oocytes	−0.049	0.726	0.278	0.211	0.118	0.416	−0.218	0.222
Number of GV Stage Oocytes	0.091	0.515	−0.015	0.948	0.251	0.079	0.244	0.171
Number of Immature Oocytes (GV + MI)	−0.002	0.989	0.297	0.179	0.260	0.069	0.091	0.613
Number of Atretic Oocytes	0.020	0.886	−0.167	0.458	0.068	0.637	−0.089	0.622
Number of Fertilized Oocytes	−0.084	0.551	0.370	0.090	0.094	0.516	−0.015	0.933
Number of Embryos Obtained	−0.080	0.571	0.361	0.099	0.094	0.516	−0.015	0.933
Maturation Rate (%)	0.122	0.385	0.232	0.298	−0.115	0.425	0.023	0.900
Fertilization Rate (%)	−0.079	0.575	−0.042	0.854	−0.072	0.619	−0.187	0.297
High-quality Embryos Rate (%)	0.028	0.844	0.066	0.771	0.077	0.595	−0.195	0.278
Cleavage Rate (%)	0.108	0.441	0.079	0.726	−0.102	0.480	0.074	0.681
Implantation Rate %	0.066	0.639	0.104	0.646	−0.029	0.840	−0.017	0.925

AMH: Anti-Müllerian Hormone, FSH: Follicle-Stimulating Hormone, GV: Germinal Vesicle, hCG: Human Chorionic Gonadotropin, PCOS: Polycystic Ovary Syndrome.

**Table 6:** FF PlGF levels between pregnant and non-pregnant women.

Group	Clinical pregnancy			Ongoing Pregnancy		
	Pregnant	Non-Pregnant	P-Value	Pregnant	Non-Pregnant	P-Value
PCOS-GnRH Agonist	144.98 ± 53.31	141.29 ± 51.05	0.928	147.37 ± 54.47	140.57 ± 50.65	0.849
PCOS-GnRH Antagonist	118.49 ± 30.62	117.24 ± 39.65	0.868	118.49 ± 30.62	117.24 ± 39.65	0.868
Control- GnRH Agonist	136.73 ± 34.35	142.06 ± 45.83	0.688	135.71 ± 31.16	141.96 ± 45.68	0.661
Control- GnRH Antagonist	120.50 ± 36.28	120.49 ± 35.40	0.953	126.88 ± 32.95	118.45 ± 36.12	0.578

PCOS: Polycystic Ovary Syndrome.

**Table 7:** Receiver Operating Characteristic (ROC) Curve to evaluate the accuracy of FF PlGF levels in predicting pregnancy rates.

Group	Clinical Pregnancy					Ongoing Pregnancy				
	AUC	Std. Error	Sig.	95% CI		AUC	Std. Error	Sig.	95% CI	
				Lower Bound	Upper Bound				Lower Bound	Upper Bound
PCOS-GnRH Agonist	0.493	0.085	0.928	0.325	0.660	0.516	0.090	0.849	0.340	0.693
PCOS-GnRH Antagonist	0.527	0.132	0.838	0.268	0.786	0.527	0.132	0.838	0.268	0.786
Controls-GnRH Agonist	0.500	0.090	1.000	0.324	0.676	0.484	0.092	0.865	0.303	0.664
Controls-GnRH Antagonist	0.491	0.113	0.936	0.270	0.712	0.570	0.106	0.556	0.363	0.777

AUC: Area under curve, PCOS: Polycystic Ovary Syndrome, 95% CI: 95% confidence interval.

## 4 Discussion

In routine practice, the starting dose of gonadotropins is individualized to assure optimum safety and efficiency of the controlled ovarian hyperstimulation (COH) in order to obtain sufficient ovarian response and reduce the risk of developing OHSS. Thus, if no previous cycles have been performed, the choice of the starting dose of gonadotropins will be based on a prediction of the ovarian response, which is built based on some patient characteristics like patient's age, ovarian reserve, day 3 FSH, and antral follicle count (AFC) [40]. In addition, the Ovarian Sensitivity Index (OSI), which is a marker link between the number of retrieved oocytes and the total administered dose of FSH, has been introduced recently to estimate ovarian sensitivity to exogenous gonadotropins, and its values negatively correlated with age and positively with AFC and the circulating levels of AMH [41]. PCOS women have higher antral follicular counts and higher levels of AMH and estradiol, which exaggerates their response and sensibility to COH [42,43] and explains the higher OSI values of this population. However, that puts PCOS women at increased risk to develop OHSS [[44], [45], [46]], so they are usually stimulated with a lower starting dose and require a lower total dose of gonadotropins during COH. In the current study, we noted that stimulating PCOS women with the GnRH agonist long protocol led to a significantly higher number of retrieved oocytes compared to the controls, and this increase in the oocytes number covered both the mature and immature oocytes. In addition, the number of fertilized oocytes and obtained embryos trend to be significantly higher in the PCOS-A group compared to the Control-A group. Interestingly, similar effects could not be detected between PCOS and controls during the GnRH antagonist protocol. Our results on the long protocol were consistent with several previous clinical studies [24,47,48]. However, they partially disagreed with the results of the prospective study of Arabzadeh et al. [49], which reported insignificant differences in the number of retrieved oocytes, maturation rate, fertilization rate, high-quality embryos rate, and implantation rate between PCOS (n = 26) and controls (n = 42) undergone the long agonist protocol. Nevertheless, Arabzadeh et al. study [49] infertility inclusion criteria for all cases included unexplained infertility, infertility due to sperm or tubal abnormalities, and endometriosis. Therefore, including women with endometriosis might have influenced the final results since endometriosis has a negative impact on IVF outcomes and is associated with lower oocyte yield, lower implantation rates, and lower pregnancy rates [50]. On the other side, our results on the GnRH antagonist protocol were consistent with the results of the prospective study of Afiat et al. [7], which could not detect any significant differences in the number of MII oocytes and MI oocytes between PCOS (n = 50) and controls (n = 50) that treated with GnRH antagonist protocol. Differently, the prospective study of Le et al. [5] showed higher numbers of retrieved oocytes and mature oocytes in the PCOS group (n = 39) compared to the control one (n = 67) during the GnRH antagonist protocol. Similarly, the retrospective study of Nikbakht et al. [6] also found a higher number of retrieved oocytes in the PCOS group. Indeed, focusing only on the number of obtained oocytes without taking into account the doses that were used for stimulation would lead to misleading results. Unfortunately, many of these studies did not mention the doses that were provided to PCOS or control women. In our study, women in the PCOS-Anta group were stimulated with (1779.55 ± 702.87) IUs and provided (17.73 ± 9.76) oocytes and (10.18 ± 5.55) MII oocytes while the Control-Anta were stimulated with (2468.18 ± 879.53) IUs and provided (16.24 ± 8.99) oocytes and (9.03 ± 5.34) MII oocytes. On the other hand, during Le et al. study [5], women in the PCOS-Anta group were stimulated with (1820.69 ± 332.06) IUs and provided (18.85 ± 9.41) oocytes and (14.97 ± 7.43) MII oocytes while in the Control-Anta were stimulated with (2005.60 ± 379.69) IUs and provided (11.48 ± 5.51) oocytes and (9.51 ± 4.7) MII oocytes. However, Nikbakht et al. [6] did report the total stimulation doses that were used in the studied groups. Although the women of the PCOS-Anta group of our study and Le et al. study [5] were stimulated with similar doses, women in the Control-Anta group of our study were stimulated with a little higher dosage of gonadotropins compared to those from Le et al. study [5] and produced higher number of retrieved oocytes. Thus, the increase in stimulators dose did not arise from a lower response to gonadotropins. Therefore, we think that stimulating the Control-Anta group in our study with a little higher dose might have prevented the differences between the two groups (PCOS-Anta vs Control-Anta) from reaching the significance level and that PCOS women would respond more aggressively to COH irrespective of the protocol used. That also can be confirmed by the fact that OSI values in our study differ significantly between PCOS women and controls independently from the protocol used. Thus, we encourage using the OSI index in clinical studies to remove the confounding effects of using different doses of gonadotropins.

Several studies raised some concerns regarding the oocyte quality of PCOS women. However, the available data are still conflicted. Niu et al. [51] suggested an association between abnormal lipid metabolism and oocyte competence, and they concluded that the high concentrations of linoleic acid and palmitoleic acid both in the plasma and in the follicular fluid of obese PCOS women might contribute to the poor pregnancy results of IVF in this population. In addition, Lai et al. [52] reported that the increased reactive oxygen species (ROS) expression levels in PCOS granulosa cells greatly induced cell apoptosis, which further affected the oocyte quality and reduced the pregnancy results. Based on our results, there were no differences in maturation rate, fertilization rate, high-quality embryos rate, cleavage rate, implantation rate, oocytes morphology, or clinical IVF/ICSI outcomes between the PCOS and the control women either during the GnRH agonist protocol or the GnRH antagonist one. This partially agrees with the prospective study of Sigala et al. [53], which also could not find any differences in the oocyte morphology, maturation rate, fertilization rate, or high-quality embryos rate between women with polycystic ovarian morphology (PCOM, n = 97) and women with normal ovarian morphology (n = 97). However, they reported a higher implantation rate, ongoing pregnancy rate, and delivery rate in the PCOM group compared to the control one. It should be taken into account that Sigala et al. [53] included both women with PCOS and women with only PCOM in the PCOM arm, and the participants were stimulated with a combination of the GnRH agonist and the GnRH antagonist protocols. In addition, the authors declared that they did not exclude low responder patients from the control group, which may explain the better clinical outcomes in the PCO group. Similarly, Afiat et al. [7] reported comparable oocyte nuclear maturity and embryo grades between PCOS and non-PCOS women during the GnRH antagonist protocol. However, time-lapse studies on embryo development ended up with contradicted results [5,[54], [55], [56]]. The retrospective study of Chappell et al. [54] showed that embryos from PCOS women (n = 64 women) displayed a faster growth rate at t7, t8, and t9 compared to controls (n = 64 women), while those from hyperandrogenic PCOS (n = 47 women) showed a faster growth rate at t5, t6, t7, t8, t9, and morula stage. Similarly, the retrospective study of Sundvall et al. [55] reported a shorter time to initiate compaction and reach the morula stage; and a shorter duration of the fourth cleavage division in the PCOS embryos compared with the non-PCOS ones, but the kinetic at other time-points were similar. On the other hand, Le et al.’s study [5] found no differences in morphokinetics or incidence of abnormalities between PCOS and non-PCOS embryos. However, the percentage of t2 stages which fell in the “optimal range” (>24 h and <28 h) was significantly lower in the PCOS group than in the control group. On the contrary, the prospective study of Wissing et al. [56] reported a significant delay in time to two pronuclei breakdown, first cleavage, and cleavage to three, four, and seven cells in embryos from hyperandrogenic PCOS (n = 25 women) compared to controls (n = 20 women). It is worth mentioning that the assessment of embryo development was carried out for a shorter duration in the Le et al. [5] (for 48 h or to the six-cell stage) and Wissing et al. [56] (to the eight-cell stage in most cases as the embryos were transferred on Day 2, and only the remaining embryos were studied until Day 5 or Day 6) studies. In addition, the studies differ in the protocol of stimulation as it was the long GnRH agonist in the Wissing et al.’s study [56], the GnRH antagonist in Le et al.’s study [5], and a combination of the two in Sundvall et al.’s study [55]. However, none of these studies could detect any differences in implantation rate, pregnancy rate, or live birth rate between PCOS and non-PCOS women, which suggests that although PCOS exaggerates ovarian response to stimulation, it does not have a detrimental impact on oocytes quality or competence. This also agrees with the results of the retrospective study of Vas et al., which reported similar rates of fertilization, implantation, and clinical pregnancy from the oocytes that were taken from PCOS donors and non-PCOS donors [57], and even if PCOS led to some minimal deviations in embryo developmental process, these deviations might not be clinically important.

In the current study, FF PLGF levels were comparable between PCOS and controls during both; the long GnRH agonist protocol and the flexible GnRH antagonist one. In addition, FF PlGF levels were negatively correlated with age and total gonadotropins dose and positively correlated with OSI in the PCOS-Anta, Control-A, and Control-Anta groups, but not in the PCOS-A group. Moreover, FF PlGF levels were positively correlated with the number of MII oocytes in the PCOS-Anta group and the number of retrieved oocytes in the Control-A group. Based on our recent work, the long GnRH agonist protocol is associated with significantly higher levels of FF PlGF compared to the flexible GnRH antagonist one both; in PCOS and normo-ovulatory women [27,28]. Therefore, the more aggressive stimulation effects of the long agonist protocol on the PCOS women might have disturbed the correlation between the FF PlGF levels and the OSI values and/or the total gonadotropins doses in the PCOS-A group. Our results partially agree with the results of the cross-sectional study of Nejabati et al. [29], which could not detect any significant differences in FF PlGF levels between poor responders (n = 30), normo-responders (n = 40), and high responders (n = 20) among non-PCOS women that undergone IVF/ICSI cycles with the long GnRH agonist protocol. However, they showed that FF PlGF levels were significantly and negatively correlated with age and total FSH dose, but not with the number of retrieved oocytes or the OSI. On the other hand, PlGF/sFlt-1 ratios were significantly and negatively correlated with age and fertilization rate while positively correlated with the number of retrieved oocytes, the number of obtained embryos, and the OSI. These differences in correlations might be related to the fact that the correlations in the Nejabati et al. [29] study were assessed among the total number of participants independent of the response classification. In addition, although there were not any differences in FF PlGF levels among various responder groups, PlGF/sFlt-1 ratios differ significantly between poor responders and high responders. Differently, the prospective cohort study of Tal et al. [26] reported significantly higher FF PlGF levels and lower FF sFlt-1 levels in PCOS women (n = 14) compared to controls (n = 14). They also demonstrated that FF PlGF levels positively correlated with the number of oocytes and the serum levels of AMH while negatively correlated with age. However, in the Tal et al. study [26], the women were simulated using both the GnRH agonist and GnRH antagonist protocols, and the correlations were evaluated among the total number of participants, i.e. they included PCOS-A, PCOS-Anta, Control-A, and Control-Anta. Regarding the differences in FF PlGF levels between PCOS and controls, although they found significantly higher levels of FF PlGF in PCOS women, while we could not detect any differences between the two populations, we do not think our results disagree with theirs. As we previously mentioned, PlGF levels differ significantly between the flexible GnRH antagonist protocol and the long GnRH agonist one, both in PCOS and normo-ovulatory women [27,28]. However, even if we compared the PCOS groups (PCOS-A + PCOS-Anta) together with the control groups (Controls-A + Controls-Anta), we could not detect any significant differences in FF PlGF levels between PCOS and controls women (data not shown). Nevertheless, the total gonadotropins doses were significantly different between (PCOS-A vs Control-A), (PCOS-Anta vs Control-Anta), and (PCOS-A + PCOS-Anta vs Control-A + Control-Anta) in our study, but not in the Tal et al. one [26]. Thus, in our opinion, it is all related to the consumed dose of gonadotropins and the OSI of the population. Since PCOS women have higher OSI and are usually considered higher responders to gonadotropins compared to controls, stimulating them with similar gonadotropins doses will produce more oocytes and require higher levels of PlGF to accomplish that response taking into account that PlGF controls ovarian angiogenesis and follicular development [14,15,58]. In addition, we do not think that this effect is specific to PCOS subjects but to all high responders, as Nejabati et al. [29] also could not detect any significant differences in FF PlGF levels between poor responders, normo-responders, and high responders when the provided gonadotropins doses were significantly lower between high responders vs poor responders and high-responders vs normo-responders.

Based on our results, FF PlGF were comparable between pregnant and non-pregnant women, both in PCOS and normo-ovulatory women, independently of the protocol used, which also had been confirmed by the ROC curve analysis. That agrees with the results of Nejabati et al. [29] on non-PCOS women during the long agonist protocol. Although PlGF levels are positively correlated with the OSI, which reflects the ovarian response to stimulation, other factors like the degree of male infertility, sperm/oocytes genetics integrity, infertility duration, and endometrial receptivity may also influence the pregnancy achievement.

## 5 Strengths, limitations, and future research

To the best of our knowledge, this is the first study that investigated the dependency of the correlations between the FF PlGF levels and the IVF/ICSI outcomes in PCOS and normo-ovulatory women on the COH protocol used. In addition, it is the first study to examine in detail the impact of PCOS on the oocyte morphology during both the long GnRH agonist protocol and the flexible GnRH antagonist one. However, our study has some limitations. First, due to the limited budget, our study was only concerned about the total FF PlGF levels and not the levels of the free form of PlGF (PlGF/sFlt-1 ratio). In addition, our study only included normo-ovulatory and PCOS women, so further research is needed to clarify whether similar correlations would be noted between FF PlGF levels and IVF/ICSI outcomes from other populations with different ovarian responses, e.g. aged women, poor responders, or endometriotic women.

## 6 Conclusions

Although PCOS exaggerates ovarian response to stimulation irrespective of the protocol used, it does not have a detrimental impact on oocytes morphology, quality, or competence. In addition, FF PlGF levels could be a marker of the ovarian response other than a predictor of pregnancy achievement during IVF/ICSI cycles independent of the PCOS pathology.

## Preprint

A preprint has previously been published [Kadoura et al., 2022] [59].

## Ethics approval

The data were adopted from studies that were performed in line with the principles of the Declaration of Helsinki. The Ethical Committee of Damascus University approved the studies’ protocols.

## Source of funding

This work was funded by 10.13039/501100020595Damascus University, Damascus, Syrian Arab Republic.

## Authors' contributions

All authors contributed to conceptualizing and designing the study. M.A. and S.K performed the clinical experiment and were responsible for the work in the field, including patients’ recruitment, sample acquisition, and data collection. S.K. performed the statistical analysis and data interpretation. S.K. drafted the manuscript, while A.N. and M.A. revised it critically for important intellectual content. All authors approved the final manuscript.

## Trail registry number

1. Name of the registry: clinicaltrials.gov
2. Unique Identifying number or registration ID: NCT04724343, NCT04727671.
3. Hyperlink to your specific registration (must be publicly accessible and will be checked): https://clinicaltrials.gov/ct2/show/NCT04724343.https://clinicaltrials.gov/ct2/show/NCT04727671.

## Guarantor

The Guarantor is Sally Kadoura who is the corresponding author of this manuscript. Email: sally.clinical@gmail.com.

## Consent to participate

A written informed consent was obtained from all individual participants included in the study.

## Availability of data and materials

The data that supports the findings are available from the corresponding author on reasonable request.

## Provenance and peer review

Not commissioned, externally peer-reviewed.

## Declaration of competing interest

The authors declare that they have no competing interests.

## Abbreviations

AFC: Antral follicle count
AMH: Anti-Müllerian hormone
AUC: Area under the curve
BPR: Biochemical Pregnancy Rate
CCR: Cycle Cancellation Rate
COCs: Cumulus oocyte complex
COH: Controlled ovarian hyperstimulation
CPR: Clinical Pregnancy Rate
FF: Follicular fluid
GnRH: Gonadotropin-releasing hormone
GV oocytes: Germinal Vesicle Oocytes
hCG: human Chorionic Gonadotropin
hMG: human Menopausal Gonadotropin
ICSI: Intra-Cytoplasmic Sperm Injection
IR: Implantation Rate
IVF: In-vitro Fertilization
MI oocytes: Metaphase I Oocytes
MII oocytes: Metaphase II Oocytes
MPR: Multiple Pregnancy Rate
OHSS: Ovarian hyperstimulation syndrome
OPR: Ongoing Pregnancy Rate
OSI: Ovarian sensitivity index
PB: Polar Body
PCOM: Polycystic ovarian morphology
PCOS: Polycystic ovary syndrome
Pesa: Percutaneous Epididymal Sperm Aspiration
PlGF: Placental growth factor
PVS: Perivitelline Space
r-FSH: recombinant Follicle-Stimulating Hormone
RPUL: Resolved Pregnancy of unknown location
ROC: Receiver operating characteristic
ROS: Reactive oxygen species
SC: subcutaneously
SER: Smooth Endoplasmic Reticulum Aggregations
sFlt-1: Soluble Fms-Like Tyrosine Kinase-1
sVEGFR-1: Soluble form of VEGF receptor-1
Tesa: Testicular Sperm Aspiration
VEGF: Vascular endothelial growth factor
ZP: Zona Pellucida
